# Associations between negative gender attitudes and eating behaviors in Chinese children and adolescents

**DOI:** 10.3389/fnut.2022.1053055

**Published:** 2023-01-04

**Authors:** Ruiyao Cao, Jiaoyan Chen, Yuanyuan Wang, Xingwang Peng, Mei Han, Keke Liu, Juan Zhang, Rongying Yao, Hui Han, Lianguo Fu

**Affiliations:** Department of Child and Adolescent Health, School of Public Health, Bengbu Medical College, Bengbu, China

**Keywords:** disliking your own gender, wanting to be opposite gender, eating behaviors, children and adolescents, negative gender attitudes

## Abstract

**Background:**

Negative gender cognitive attitudes (disliking one’s own gender or wanting to be the opposite gender) and unhealthy eating behaviors have become common in Chinese children and adolescents. The aim of this study was to analyze the associations between negative gender attitudes and eating behaviors among Chinese children and adolescents.

**Methods:**

Primary and secondary school students aged 8–15 years were selected as participants using a stratified cluster random sampling method. The self-designed questionnaire was used to investigate the participants’ negative gender cognitive attitudes. Eating frequency questionnaire was used to investigate participants’ eating behaviors. Under the leading reading of standardized training investigators, the questionnaire for children aged 8–15 years was completed by themselves in the form of centralized filling.

**Results:**

A total of 6.5% [43/657, boys: 6.1% (21/347), girls: 7.1% (22/310)] of children disliked their own gender, 8.8% [58/657, boys: 5.5% (19/347), girls: 12.6% (39/310)] of children wanted to be of the opposite gender, and the proportion of girls with negative gender attitudes was higher than that of boys (*P* < 0.05). Boys who disliked their own gender or wanted to be the opposite gender had higher frequencies of unhealthy eating behaviors and lower frequencies of healthy eating behaviors than boys who liked their own gender or did not want to be the opposite gender (*P* < 0.05). Girls who disliked their own gender or wanted to be the opposite gender had higher frequencies of protein eating behaviors than girls who liked their own gender or did not want to be the opposite gender (*P* < 0.05). There was a significant interaction between disliking one’s own gender and wanting to be the opposite gender in midnight snack eating among boys (*P* < 0.05) and in carbonated drink and high protein eating behaviors among girls (*P* < 0.05).

**Conclusion:**

Boys with negative gender cognitive attitudes express more unhealthy eating behaviors and fewer healthy eating behaviors; girls with negative gender cognitive attitudes exhibit more protein eating behaviors.

## Introduction

The gender role is the construction of gender attitudes, behaviors, cognitions and emotions in a specific cultural background (1). Most children’s gender experience of being male or female is in line with the gender assigned at birth, but inconsistencies can lead to gender dysphoria (2). Children who have a strong desire to be another gender are defined as children with gender dysphoria (3, 4). A study among Dutch children showed that 1.4% of boys wished to be of the opposite gender, and 2.0% of girls wished to be of the opposite gender (5). Recent studies suggested that the prevalence of a self-reported transgender identity in children and adolescents ranges from 0.5 to 1.3% and is more prominent among girls than boys (6). Compared with children without gender dysphoria, children with gender dysphoria have a higher risk of eating disorders (7, 8). Some studies in Canadian adolescents showed that 5% of adolescents with gender dysphoria had eating disorders (9). Transgender adolescents participate in negative eating behaviors to change their appearance or modify their bodies to adapt to their gender identity (10). The increase in disordered eating behaviors will lead to higher dissatisfaction with their bodies (11) and contribute to adolescents becoming sexual minorities. Gender role orientation plays an important role in adolescent eating disorders (12). Some children and adolescents may not be defined as gender dysphoric, but they may dislike their gender or want to be the opposite gender. There have been few reports on whether these negative gender attitudes are related to eating behaviors.

Adolescence is a window of opportunity to develop healthy dietary patterns and nutritional status, which may have lasting effects on future health (13). The unhealthy dietary characteristics of adolescents include a low intake of fruits and vegetables and a high intake of energy-intensive and nutrient-poor foods, such as carbonated drinks and western fast food (14). Micronutrient deficiencies are prevalent among adolescents, with profound effects on their quality of life, risk of premature death, and the health of their offspring (15). Dietary quality plays a key role in preventing these forms of adolescent malnutrition. High intake of fruits and vegetables has been associated with increased blood vitamin concentrations in European adolescents (16), while low intake is a major contributor to the global burden of disease, particularly non-communicable diseases (17). Carbonated drinks usually contain a small amount of nutrients and sugar. Drinking sugary drinks is closely related to obesity and type 2 diabetes in adolescents (18). Adolescents’ fast food consumption is associated with metabolic markers of obesity, diabetes and cardiovascular disease (19).

Healthy eating behaviors are important to develop health among children, and negative gender attitudes may play an important role in eating behaviors. The purpose of this study was to analyze the associations between negative gender attitudes and eating behaviors, which is a new perspective to promote healthy eating behaviors among children and adolescents.

## Materials and methods

### Participants

A total of 692 students (369 boys and 318 girls) aged 8–15 years were selected from two 9-year compulsory education schools using the stratified cluster sampling method. There were 657 effective samples (347 boys, 310 girls), and the effective rate was 99.0%. The project was approved by the Medical Research Ethics Committee of Bengbu Medical College [(2015) No. 003], and participants’ guardians signed informed consent forms. This study was conducted in accordance with the Declaration of Helsinki.

### Measurement

Medical staff who receive standardized training were recruited as surveyors. The participants were asked to have an empty stomach, wear light clothes, and be barefoot. Height was measured using a mechanical height meter to the nearest 0.1 cm. Weight was measured using an electronic body weight meter to the nearest 0.1 kg. Waist circumference (WC) was measured to the nearest 0.1 cm using a nylon tape scale. WC is the circumference of the waist along a horizontal line at 1 cm above the navel. BMI = Weight (kg)/Height (m)^2^; Waist-to-height ratio (WHtR) = WC (cm)/Height (cm). The students were divided into overall overweight/obese and non-overweight/obese groups according to the sex-age BMI standard (20). According to the standard of WHtR ≥ 0.46, children and adolescents were divided into abdominal obesity and non-abdominal obesity groups (21). Under the leading reading of standardized training investigators, the questionnaires of eating behaviors and negative gender attitude for children aged 8–15 years were completed by themselves in the form of centralized filling.

### Eating behaviors survey

An eating frequency questionnaire was used to investigate the frequencies of dining out, midnight snacks, fried food, high-calorie snacks, preserved vegetables, western fast food, carbonated drink, fish meat, shrimps, eggs, milk, fresh vegetables, fruits, and breakfast. The frequency of each eating behavior was assigned as 1 time/day = 7, 4–6 times/week = 5, 1–3 times/week = 2, 1 time/2 weeks = 0.5, 1 time/month = 0.25, and never = 0 (22). The higher the score, the higher the frequency of eating behaviors. The Cronbach coefficient of the questionnaire was 0.630. Exploratory factor analysis (EFA) was used to extract 3 common factors from 14 eating behaviors by orthogonal rotation with maximum variance, including unhealthy eating behavior, healthy eating behavior and protein eating behavior. The unhealthy eating behavior factor included dining out and consuming midnight snacks, fried food, high-calorie snacks, preserved vegetables, western fast food, and carbonated drink; the healthy eating behavior factor included eggs, milk, fresh vegetables, fruits and breakfast; and the protein eating behavior factor included fish, meat and shrimp. The KMO test value of factor analysis was 0.770, and Bartlett’s sphericity test showed a statistically significant difference (χ^2^ = 1465.198, *P* < 0.001) (see Table 1 for details).

**TABLE 1:** Exploratory factor analysis of eating behavior frequency.

Eating behaviors	Load of unhealthy eating behavior factor	Load of healthy eating behavior factor	Load of protein eating behavior factor
Dine out	0.567		
Midnight snack	0.489		
Fried food	0.671		
High-calorie snacks	0.696		
Preserved vegetables	0.395		
Western fast food	0.631		
Carbonated drink	0.578		
Fish meat			0.849
Shrimps			0.827
Eggs		0.636	
Milk		0.704	
Fresh vegetables		0.709	
Fruits		0.751	
Breakfast		0.492	
Eigenvalue	2.453	2.279	1.638
Contribution rate (%)	17.519	16.282	11.700
Cumulative contribution rate (%)	17.519	33.801	45.501

### Negative gender attitude survey

The self-designed questionnaire was developed to investigate children and adolescents’ negative gender attitudes. Question 1 (Disliking your own gender): Do you like your own gender? Answer: Like, Not completely like, Dislike. Disliking your own gender was divided into two ranks: “Yes” (“Not completely like” or “dislike”) and “No” (“Like”). Question 2 (Wanting to be the opposite gender): Do you want to be a member of the opposite gender? Answer: Not wanting, Not completely wanting, Wanting (23). Wanting to be the opposite gender was divided into two ranks: “Yes” (“Wanting” or “Not completely wanting”) and “No” (“Not wanting”). The Cronbach’s coefficient of the questionnaire was 0.728.

### Statistical analyses

All analyses were conducted using IBM SPSS 23.0 software. The quantitative data are described as the mean ± SD, and count data are described as the rate or proportion (%). Two independent sample *t*-tests were used to compare the differences in quantitative variables between the two groups. The *chi*-square test was used to compare the differences in qualitative variables between the two groups. After adjusting for age and body type, multiple linear regression was used to analyze the relationships between negative gender attitudes and eating behaviors. *P* < 0.05 was considered statistically significant.

## Results

### Differences in age, eating behaviors, gender attitudes, and body shape between boys and girls

**FIGURE 1:**
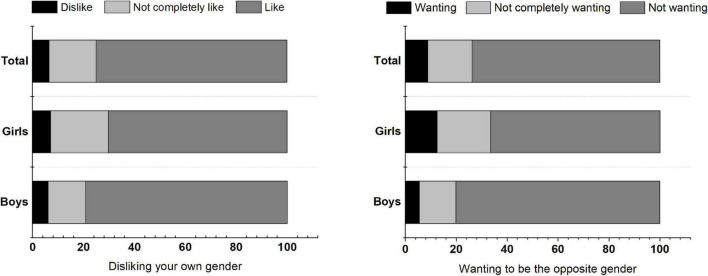
Proportion of disliking your own gender and wanting to be the opposite gender among children and adolescents.

A total of 657 adolescents aged 8–15 years were included in this study, including 347 boys and 310 girls. The proportion of children disliking their own gender was 6.5% [43/657, boys: 6.1% (21/347), girls: 7.1% (22/310)], and the proportion of children who wanted to be of the opposite gender was 8.8% [58/657, boys: 5.5% (19/347), girls: 12.6% (39/310)] (see Figure 1 for details). The proportion of girls with the above negative gender attitudes was significantly higher than those of boys (*P* < 0.05). The proportion of children with abdominal obesity and overall overweight or obesity was 29.8% [196/657, boys: 33.1% (115/347), girls: 26.1% (81/310)] and 31.5% [207/657, boys: 34.3% (119/347), girls: 28.4% (88/310)], respectively, and was higher in boys than in girls (*P* < 0.05). There was no significant difference in age between boys and girls (*P* > 0.05). The frequencies of consuming breakfast, midnight snacks, preserved vegetables, western fast food, carbonated drinks, fish, meat, and shrimp were higher in boys than in girls, as well as unhealthy eating behaviors and high protein eating behaviors (*P* < 0.05). There were no significant differences between boys and girls in the frequencies of dining out, eating fried food, high-calorie snacks, fresh vegetables and fruits (*P* > 0.05) (see Table 2 for details).

**TABLE 2:** Comparisons of differences in age, eating behaviors, gender attitude, and body shape between boys and girls.

Variables	Boys (*n* = 347)	Girls (*n* = 310)	t/X^2^	*P*
Age	11.3 ± 1.8	11.5 ± 1.80	0.17	0.677
Dine out	2.2 ± 2.3	1.8 ± 2.1	3.64	0.057
Breakfast	6.1 ± 1.8	5.8 ± 2.1	8.92	0.003
Fried food	2.0 ± 2.1	1.9 ± 2.1	0.10	0.755
High-calorie snacks	2.0 ± 2.3	2.0 ± 2.2	0.55	0.461
Midnight snack	1.9 ± 2.6	1.4 ± 2.5	8.22	0.004
Western fast food	1.0 ± 1.8	0.8 ± 1.5	10.82	0.001
Carbonated drink	1.4 ± 2.0	0.8 ± 1.5	37.33	0.001
Preserved vegetables	2.0 ± 2.3	1.6 ± 2.1	4.97	0.026
Fish meat	2.7 ± 2.3	2.1 ± 2.2	9.61	0.002
Shrimps	1.7 ± 2.2	1.5 ± 1.9	5.88	0.016
Eggs	4.8 ± 2.5	4.6 ± 2.6	4.42	0.036
Fresh vegetables	5.3 ± 2.3	5.5 ± 2.2	0.43	0.512
Fruits	5.6 ± 2.0	5.5 ± 2.2	1.74	0.188
Unhealthy eating behavior factor	0.1 ± 1.05	−0.11 ± 0.93	4.22	0.040
Healthy eating behavior factor	0.04 ± 1.00	−0.05 ± 1.00	0.15	0.694
Protein eating behavior factor	0.11 ± 1.05	−0.12 ± 0.93	5.94	0.015
disliking your own gender			6.97	0.008
Yes	72 (20.7)	92 (29.7)		
No	275 (79.3)	218 (70.3)		
Wanting to be the opposite gender			15.76	0.000
Yes	69 (19.9)	104 (33.5)		
No	278 (80.1)	206 (66.5)		
Overall overweight or obesity			2.65	0.104
Yes	119 (34.3)	88 (28.4)		
No	228 (65.7)	222 (71.6)		
Abdominal obesity			3.85	0.050
Yes	115 (33.1)	81 (26.1)		
No	232 (66.9)	229 (73.9)		

### Comparisons of eating behaviors and negative gender attitudes between children and adolescents with different body shapes

The frequency of drinking carbonated drinks among girls with overall overweight or obesity was higher than that of girls with non-overall overweight or obesity, but the frequencies of eating midnight snacks and eggs among girls with non-overall overweight or obesity were higher than those of girls with overall overweight or obesity. The frequencies of dining out, eating western fast food and high protein eating behaviors among abdominally obese boys were higher than those in non-abdominally obese boys (*P* < 0.05). The frequency of drinking carbonated drinks among girls with abdominal obesity was higher than that in girls with non-abdominal obesity (*P* < 0.05), but the frequencies of eating fish, meat and eggs among girls with non-abdominal obesity were higher than those in girls with abdominal obesity (*P* < 0.05) (see Table 3 for details).

**TABLE 3:** Comparisons of differences in eating behaviors, negative gender attitude between children and adolescents with different body shapes.

Variables	Overall overweight or obesity		t/X^2^	*P*	Abdominal obesity		t/X^2^	*P*
	Yes (*n* = 207)	No (*n* = 450)			Yes (*n* = 196)	No (*n* = 461)		
**Boys**								
Dine out	2.5 ± 2.3	2.1 ± 2.3	0.60	0.441	2.7 ± 2.4	2.0 ± 2.2	5.31	0.022
Breakfast	6.0 ± 1.8	6.2 ± 1.7	2.53	0.113	6.0 ± 1.9	6.2 ± 1.7	3.68	0.056
Midnight snack	1.7 ± 2.5	1.9 ± 2.7	0.34	0.559	1.8 ± 2.6	1.9 ± 2.6	0.15	0.701
Fried food	2.2 ± 2.2	1.9 ± 2.1	0.30	0.583	2.1 ± 2.1	1.9 ± 2.1	0.07	0.800
High-calorie snacks	1.9 ± 2.2	2.1 ± 2.3	0.05	0.817	1.9 ± 2.3	2.1 ± 2.3	0.01	0.920
Western fast food	1.1 ± 1.9	1.0 ± 1.7	0.75	0.388	1.2 ± 2.0	0.9 ± 1.7	5.64	0.018
Carbonated drink	1.5 ± 2.0	1.4 ± 2.0	0.05	0.822	1.6 ± 2.2	1.4 ± 1.9	2.82	0.094
Preserved vegetables	2.1 ± 2.3	2.0 ± 2.3	0.45	0.501	2.1 ± 2.3	2.0 ± 2.3	0.21	0.650
Fish meat	2.6 ± 2.4	2.7 ± 2.2	0.76	0.384	2.7 ± 2.4	2.7 ± 2.2	1.45	0.229
Shrimps	1.9 ± 2.3	1.7 ± 2.2	0.03	0.870	2.0 ± 2.4	1.6 ± 2.1	2.59	0.109
Eggs	4.6 ± 2.4	4.9 ± 2.5	0.76	0.384	4.7 ± 2.4	4.8 ± 2.5	0.706	0.401
Milk	5.5 ± 2.1	5.5 ± 2.2	0.10	0.758	5.4 ± 2.2	5.6 ± 2.1	0.14	0.709
Fresh vegetables	5.1 ± 2.3	5.4 ± 2.3	0.01	0.926	5.1 ± 2.3	5.4 ± 2.2	0.07	0.799
Fruits	5.6 ± 1.9	5.6 ± 2.1	0.74	0.389	5.6 ± 2.0	5.7 ± 2.1	0.37	0.546
Unhealthy eating behavior factor	0.14 ± 0.99	0.07 ± 1.08	0.98	0.324	0.18 ± 1.05	0.05 ± 1.05	0.00	0.996
Healthy eating behavior factor	−0.04 ± 0.98	0.08 ± 1.01	0.17	0.678	−0.05 ± 1.03	0.09 ± 0.98	0.10	0.759
Protein eating behavior factor	0.13 ± 1.12	0.09 ± 1.01	1.78	0.183	0.19 ± 1.15	0.07 ± 0.99	5.60	0.019
Disliking your own gender			1.44	0.230			2.09	0.148
Yes	29 (24.4)	43 (18.9)			29 (25.2)	43 (18.5)		
No	90 (75.6)	185 (81.1)			86 (74.8)	189 (81.5)		
Wanting to be the opposite gender			0.01	0.924			0.11	0.746
Yes	24 (20.2)	45 (19.7)			24 (20.9)	45 (19.4)		
No	95 (79.8)	183 (80.3)			91 (79.1)	187 (80.6)		
**Girls**								
Dine out	1.7 ± 2.2	1.8 ± 2.1	0.11	0.746	1.7 ± 2.1	1.8 ± 2.2	0.002	0.965
Breakfast	5.7 ± 2.2	5.9 ± 2.0	1.10	0.295	5.5 ± 2.2	5.9 ± 2.0	2.50	0.115
Midnight snack	0.9 ± 2.1	1.6 ± 2.6	13.47	0.000	1.1 ± 2.3	1.5 ± 2.5	3.14	0.077
Fried food	1.4 ± 1.8	2.0 ± 2.2	2.43	0.120	1.6 ± 1.9	2.0 ± 2.2	1.84	0.176
High-calorie snacks	2.1 ± 2.3	2.0 ± 2.1	0.84	0.361	1.9 ± 2.2	2.1 ± 2.2	0.27	0.604
Western fast food	0.7 ± 1.3	0.8 ± 1.6	0.54	0.462	0.7 ± 1.2	0.8 ± 1.6	1.55	0.214
Carbonated drink	1.0 ± 1.8	0.8 ± 1.4	7.00	0.009	1.1 ± 1.8	0.7 ± 1.4	7.93	0.005
Preserved vegetables	1.5 ± 2.1	1.6 ± 2.1	0.32	0.573	1.5 ± 2.0	1.6 ± 2.1	0.70	0.402
Fish meat	2.1 ± 2.2	2.1 ± 2.2	0.21	0.645	2.0 ± 1.9	2.2 ± 2.3	6.31	0.013
Shrimps	1.7 ± 2.0	1.4 ± 1.9	0.40	0.530	1.7 ± 1.8	1.4 ± 2.0	0.68	0.411
Eggs	4.0 ± 2.7	4.9 ± 2.6	5.40	0.021	4.0 ± 2.8	4.8 ± 2.5	12.53	0.000
Milk	5.3 ± 2.4	5.3 ± 2.2	0.75	0.388	5.5 ± 2.2	5.3 ± 2.3	2.44	0.119
Fresh vegetables	5.4 ± 2.3	5.5 ± 2.1	1.79	0.182	5.7 ± 2.1	5.4 ± 2.2	0.39	0.535
Fruits	5.3 ± 2.2	5.5 ± 2.1	0.82	0.367	5.2 ± 2.2	5.6 ± 2.1	0.63	0.430
Unhealthy eating behavior factor	−0.23 ± 0.94	−0.06 ± 0.92	0.22	0.639	−0.19 ± 0.92	−0.08 ± 0.94	0.002	0.967
Healthy eating behavior factor	−0.19 ± 1.04	0.01 ± 0.99	0.14	0.706	−0.16 ± 1.03	−0.01 ± 0.99	0.12	0.732
Protein eating behavior factor	0.00 ± 0,96	−0.16 ± 0.91	0.32	0.574	−0.07 ± 0.81	−0.14 ± 0.97	2.63	0.106
Disliking your own gender			0.06	0.807			0.31	0.579
Yes	27 (30.7)	65 (29.3)			26 (32.1)	66 (28.8)		
No	61 (69.3)	157 (70.7)			55 (67.9)	163 (71.2)		
Wanting to be the opposite gender			0.45	0.501			0.35	0.552
Yes	27 (30.7)	77 (34.7)			25 (30.9)	79 (34.5)		
No	61 (69.3)	145 (65.3)			56 (69.1)	150 (65.5)		

### Comparisons of eating behaviors of children and adolescents who dislike their own gender and who like their own gender

The results showed that boys who disliked their own gender had higher frequencies of dining out and eating midnight snacks, high-calorie snacks, western fast food, preserved vegetables, unhealthy eating behaviors and protein eating behaviors than boys who liked their own gender. Girls who liked their own gender (*P* < 0.05) consumed fruits and fresh vegetables less frequently and expressed fewer healthy eating behaviors than both boys who liked their own gender and girls who liked their own gender (except for fruits) (*P* < 0.05). Girls who disliked their own gender dined out and consumed western fast food, carbonated drinks, fish, meat, and shrimp more frequently and exhibited more protein eating behaviors than girls who liked their own gender (*P* < 0.05). However, there were no significant differences in dining out or consuming western fast food, carbonated drinks, fish, meat, shrimp and protein eating behaviors between girls who disliked their own gender and boys who liked their own gender (see Table 4 for details).

**TABLE 4:** Comparisons of differences in eating behaviors between children and adolescents with disliking their own gender and liking their own gender.

Variables	Disliking your own gender in boys		t/X^2^	*P*	Disliking your own gender in girls		t/X^2^	*P*
	Yes (*n* = 72)	No (*n* = 275)			Yes (*n* = 92)	No (*n* = 218)		
Dine out	2.8 ± 2.5^a^	2.1 ± 2.2	0.25	0.013	2.1 ± 2.4	1.7 ± 2.0^a^	4.49	0.035
Breakfast	6.0 ± 2.0	6.2 ± 1.7	2.92	0.089	5.8 ± 2.0	5.8 ± 2.1	0.00	0.992
Midnight snack	2.6 ± 2.9^a^	1.7 ± 2.5	9.22	0.003	1.3 ± 2.5	1.4 ± 2.5^a^	0.13	0.720
Fried food	2.3 ± 2.3	1.9 ± 2.1	0.50	0.480	2.1 ± 2.2	1.8 ± 2.1	0.61	0.435
High-calorie snacks	2.4 ± 2.6^a^	1.9 ± 2.2	8.19	0.004	2.3 ± 2.2	1.9 ± 2.1^a^	0.13	0.722
Western fast food	1.5 ± 2.3^a^	0.9 ± 1.6	22.76	<0.001	1.0 ± 1.9	0.7 ± 1.3^a^	13.96	<0.001
Carbonated drink	1.7 ± 2.1	1.4 ± 2.0	0.35	0.553	1.1 ± 1.8	0.7 ± 1.4	9.17	0.003
Preserved vegetables	2.6 ± 2.6^a^	1.9 ± 2.2	11.01	0.001	1.8 ± 2.3	1.4 ± 2.0^a^	2.11	0.148
Fish meat	2.7 ± 2.5	2.7 ± 2.3	0.71	0.399	2.5 ± 2.5	2.0 ± 2.0	13.50	<0.001
Shrimps	1.9 ± 2.4	1.7 ± 2.2	0.52	0.470	1.8 ± 2.3	1.4 ± 1.7	9.63	0.002
Eggs	4.5 ± 2.6	4.9 ± 2.5	0.98	0.324	4.4 ± 2.7	4.7 ± 2.6	0.52	0.470
Milk	5.2 ± 2.4	5.6 ± 2.1	2.94	0.087	5.1 ± 2.3	5.4 ± 2.3	0.04	0.851
Fresh vegetables	4.5 ± 2.7^a^	5.5 ± 2.1	18.55	<0.001	5.4 ± 2.3	5.5 ± 2.1^a^	0.89	0.346
Fruits	5.3 ± 2.4	5.7 ± 1.9	10.21	0.002	5.4 ± 2.3	5.5 ± 2.1	0.41	0.522
Unhealthy eating behavior factor	0.43 ± 1.29^a^	0.01 ± 0.96	8.12	0.005	0.05 ± 1.01	-0.17 ± 0.89^a^	0.17	0.679
Healthy eating behavior factor	-0.21 ± 1.19^a^	0.11 ± 0.93	7.62	0.006	-0.12 ± 1.06	-0.02 ± 0.98^a^	1.58	0.210
Protein eating behavior factor	0.12 ± 1.20^a^	0.10 ± 1.01	4.58	0.033	0.05 ± 1.13	-0.19 ± 0.83^a^	14.07	<0.001
Wanting to be the opposite gender			110.43	<0.001			123.09	<0.001
Yes	46 (63.9)	23 (8.4)			73 (79.3)	31 (14.2)		
No	26 (36.1)	252 (91.6)			19 (20.7)	187 (85.8)		

^a^Boys with disliking their own gender (including not completely like or dislike) vs. girls with liking their own gender, *P* < 0.05; ^b^girls with disliking their own gender (including not completely like or dislike) vs. boys with liking their own gender, *P* < 0.05.

### Comparisons of eating behaviors of children and adolescents who want to be the opposite gender and those who do not

The results showed that boys who wanted to be the opposite gender ate western fast food, high-calorie snacks, and preserved vegetables more frequently and exhibited more unhealthy eating behaviors than boys who did not want to be the opposite gender. Girls who did not want to be the opposite gender (*P* < 0.05) ate eggs, fresh vegetables, and fruits less frequently and exhibited fewer healthy eating behaviors than boys who did not want to be the opposite gender (*P* < 0.05) and ate fresh vegetables and fruits less frequently than girls who did not want to be the opposite gender (*P* < 0.05). There were no significant differences in the frequencies of eating eggs and healthy eating behaviors between boys who wanted to be of the opposite gender and girls who did not want to be of the opposite gender (*P* > 0.05). Girls who wanted to be the opposite gender ate shrimp, fish, and meat more frequently and expressed more high protein eating behaviors than girls who did not want to be the opposite gender (*P* < 0.05) and ate eggs less frequently than girls who did not want to be the opposite gender and boys who did not want to be the opposite gender (*P* < 0.05). There were no significant differences in the frequencies of shrimp, fish, and meat consumption or high protein eating behaviors between girls who wanted to be of the opposite gender and boys who did not want to be of the opposite gender (*P* > 0.05) (see Table 5 for details).

**TABLE 5:** Comparisons of differences in eating behaviors between children and adolescents with wanting to be opposite gender and no wanting to be opposite gender.

Variables	Wanting to be the opposite gender in boys		t/X^2^	*P*	Wanting to be the opposite gender in girls		t/X^2^	*P*
	Yes (*n* = 69)	No (*n* = 278)			Yes (*n* = 104)	No (*n* = 206)		
Dine out	2.5 ± 2.4	2.2 ± 2.3	1.22	0.270	1.7 ± 2.0	1.9 ± 2.2	0.87	0.351
Breakfast	6.1 ± 1.9	6.2 ± 1.7	0.80	0.371	5.9 ± 1.9	5.8 ± 2.1	1.32	0.252
Midnight snack	2.3 ± 2.8^a^	1.7 ± 2.6	2.29	0.131	1.2 ± 2.3	1.5 ± 2.5^a^	2.87	0.094
Fried food	2.0 ± 2.3	2.0 ± 2.1	0.85	0.356	2.0 ± 2.1	1.8 ± 2.1	0.00	0.973
High-calorie snacks	2.2 ± 2.6^a^	2.0 ± 2.2	7.52	0.006	2.0 ± 2.1	2.1 ± 2.2^a^	2.23	0.136
Western fast food	1.3 ± 2.2^a^	0.9 ± 1.7	9.92	0.002	0.7 ± 1.4	0.8 ± 1.6^a^	1.05	0.307
Carbonated drink	1.7 ± 2.3	1.4 ± 1.9	3.75	0.054	1.0 ± 1.7	0.8 ± 1.4	3.49	0.063
Preserved vegetables	2.3 ± 2.5^a^	1.9 ± 2.2	4.80	0.029	1.6 ± 2.1	1.5 ± 2.1^a^	0.11	0.738
Fish meat	2.6 ± 2.3	2.7 ± 2.3	0.17	0.684	2.3 ± 2.4	2.0 ± 2.1	5.20	0.023
Shrimps	1.7 ± 2.3	1.7 ± 2.2	0.07	0.791	1.8 ± 2.3	1.4 ± 1.7	8.23	0.004
Eggs	4.5 ± 2.7	4.9 ± 2.4^b^	4.57	0.033	4.4 ± 2.8^b^	4.7 ± 2.6	4.13	0.043
Milk	5.4 ± 2.1	5.5 ± 1.2	0.42	0.517	5.2 ± 2.3	5.4 ± 2.3	0.03	0.855
Fresh vegetables	5.0 ± 2.6^a^	5.4 ± 2.2	4.56	0.033	5.4 ± 2.2	5.5 ± 2.2^a^	0.25	0.616
Fruits	5.1 ± 2.5^a^	5.8 ± 1.9	14.00	<0.001	5.4 ± 2.3	5.5 ± 2.1^a^	0.76	0.384
Unhealthy eating behavior factor	0.27 ± 1.31^a^	0.05 ± 0.97	11.64	0.001	-0.14 ± 0.90	-0.09 ± 0.90^a^	0.54	0.462
Healthy eating behavior factor	-0.12 ± 1.17	0.08 ± 0.95	4.17	0.042	-0.09 ± 1.04	-0.02 ± 0.99	0.67	0.415
Protein eating behavior factor	0.10 ± 1.15	0.11 ± 1.02	2.12	0.147	0.02 ± 0.90	-0.19 ± 0.85	6.32	0.012

^a^Boys who wanted to be opposite gender (including wanting and not completely wanting) vs. girls who did not want to be opposite gender, *P* < 0.05; ^b^girls who wanted to be opposite gender (including wanting and not completely wanting) vs. boys who did not want to be opposite gender, *P* < 0.05.

### Interactions between disliking your own gender and wanting to be the opposite gender in eating behaviors

**FIGURE 2:**
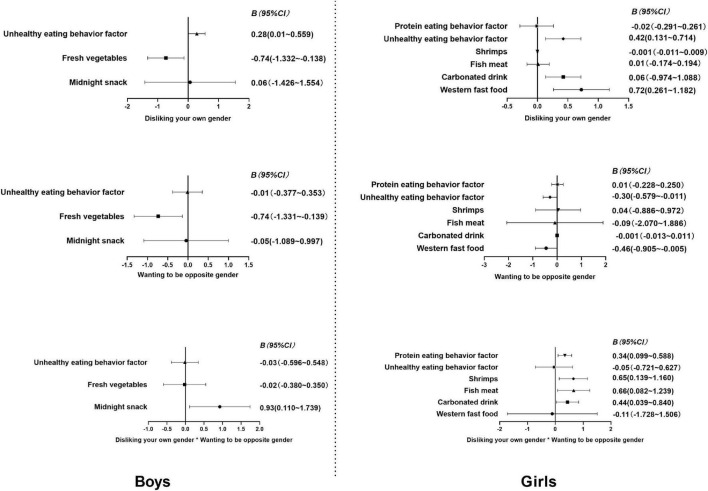
95% CI of association coefficients between eating behaviors and negative gender attitude.

After adjusting for age, overall obesity and abdominal obesity, each eating behavior was treated as a dependent variable. Disliking one’s own gender (yes = 1, no = 0), wanting to be the opposite gender (yes = 1, no = 0), and the interaction item between disliking one’s own gender and wanting to be the opposite gender were used as independent variables. Multiple linear regressions were performed using a stepwise method. The results showed that disliking one’s own gender positively correlated with unhealthy eating behavior and negatively correlated with eating fresh vegetables in boys (*P* < 0.05). There was a significant interaction between disliking one’s own gender and wanting to be of the opposite gender predicting midnight snack consumption (*P* < 0.05). Disliking one’s own gender positively correlated with eating western fast food and unhealthy eating behavior in girls (*P* < 0.05). Wanting to be the opposite gender negatively correlated with eating western fast food and unhealthy eating behavior in girls (*P* < 0.05). There were positive interactions between disliking one’s own gender and wanting to be the opposite gender that predicted consumption of carbonated drinks, fish, meat, and shrimp and high protein eating among girls (*P* < 0.05) (see Table 6 and Figure 2 for details).

**TABLE 6:** The results of multiple linear regression on associations between eating behaviors and negative gender attitude after adjusting for age, overall obesity and abdominal obesity.

Gender	Dependent variables	Independent variables	*B*	SE	Beta	*t*	*P*
Boys	Midnight snack	Disliking your own gender	0.06	0.76	0.45	0.04	0.395
		Wanting to be opposite gender	-0.05	-0.53	0.60	-0.03	0.384
		Disliking your own gender × wanting to be opposite gender	0.93	0.41	0.12	2.23	0.026
	Fresh vegetables	Disliking your own gender	-0.74	0.30	-0.13	-2.42	0.016
		Wanting to be opposite gender	0.05	0.78	0.44	0.04	0.681
		Disliking your own gender × wanting to be opposite gender	-0.02	-0.29	0.77	-0.02	0.411
	Unhealthy eating behavior factor	Disliking your own gender	0.28	0.14	0.11	2.04	0.043
		Wanting to be opposite gender	-0.01	-0.19	0.85	-0.01	0.681
		Disliking your own gender × wanting to be opposite gender	-0.02	-0.18	0.85	-0.01	0.411
Girls	Western fast food	Disliking your own gender	0.72	0.23	0.22	3.08	0.002
		Wanting to be opposite gender	-0.46	0.23	-0.14	-1.99	0.048
		Disliking your own gender × wanting to be opposite gender	-0.11	-0.83	0.41	-0.05	0.170
	Carbonated drink	Disliking your own gender	0.06	0.53	0.60	0.03	0.268
		Wanting to be opposite gender	-0.00	-0.01	1.00	0.00	0.385
		Disliking your own gender × wanting to be opposite gender	0.44	0.20	0.12	2.16	0.031
	Fish meat	Disliking your own gender	0.01	0.09	0.93	0.01	0.268
		Wanting to be opposite gender	-0.09	-1.01	0.31	-0.06	0.385
		Disliking your own gender × wanting to be opposite gender	0.66	0.29	0.13	2.25	0.025
	Shrimps	Disliking your own gender	-0.00	-0.01	1.00	0.00	0.268
		Wanting to be opposite gender	0.04	0.47	0.64	0.03	0.385
		Disliking your own gender × wanting to be opposite gender	0.65	0.26	0.14	2.51	0.013
	Unhealthy eating behavior factor	Disliking your own gender	0.42	0.15	0.21	2.86	0.005
		Wanting to be opposite gender	-0.30	0.14	-0.15	-2.04	0.042
		Disliking your own gender × wanting to be opposite gender	-0.05	-0.34	0.73	-0.02	0.170
	Protein eating behavior factor	Disliking your own gender	-0.02	-0.14	0.89	-0.01	0.268
		Wanting to be opposite gender	0.01	0.12	0.90	0.01	0.385
		Disliking your own gender × wanting to be opposite gender	0.34	0.12	0.16	2.76	0.006

Disliking your own gender × wanting to be opposite gender: Interaction item between disliking one’s own gender and wanting to be the opposite gender.

## Discussion

It is common for children and adolescents to have negative gender attitudes (disliking their own gender or wanting to be the opposite gender) and unhealthy eating behaviors, and adolescence is an important stage in the formation of gender role orientation and healthy eating behaviors. This study reported the characteristics of negative gender attitudes and eating behavior among Chinese children and adolescents and revealed the relationships between negative gender attitudes and eating behaviors for the first time, to our knowledge. The study found that boys who did not like their gender or wanted to be the opposite gender had more unhealthy eating behaviors, while they had fewer healthy eating behaviors; Girls who did not like their gender or wanted to be the opposite gender had more protein eating behaviors.

The results of this study showed that 6.5% of children disliked their own gender, 8.8% of children wanted to be of the opposite gender, and the proportion of girls with negative gender attitudes was higher than that of boys. Steensma et al. (24) estimated that the incidence of persistent gender dysphoria from childhood to adolescence ranges from 2 to 27%. Cohen-Kettenis et al. (25) confirmed that girls had a higher tolerance for transgender behaviors. Zhang et al. (26) reported that girls were more dissatisfied with their gender than boys. Social culture may play an important role in negative gender attitudes. Zucker et al. (27) found that boys’ feminine behaviors were less accepted than girls’ masculine behaviors. This study showed that the proportion of children with abdominal obesity and overall obesity was 29.8 and 31.5%, respectively, and was higher in boys than in girls, which may be related to the fact that boys ate breakfast, midnight snacks, preserved vegetables, western fast food, carbonated drinks, fish, meat, and shrimp more frequently and expressed more unhealthy dietary behaviors and more protein dietary behaviors than girls. A study found that boys were twice as likely to be overweight as girls, and that boys’ less healthy eating habits were associated with higher daily intake of protein-rich foods (28). Previous studies have proven that girls are more likely than boys to skip midnight snacks, eat more fruits and drink fewer carbonated drinks (29).

We found that boys who disliked their own gender or who wanted to be the opposite gender expressed more unhealthy eating behaviors and fewer healthy eating behaviors; girls who disliked their own gender had more frequent unhealthy eating behaviors. Becker et al. (30) reported that people with gender dysphoria suffered from eating disorders due to dissatisfaction with their gender-related body parts, inner distress and inability to accept them. Studies have shown that male sexual minorities may have lower diet quality and worse eating habits than non-minorities (31). Transgender individuals conceal or demonstrate their specific gender characteristics by unhealthy eating behaviors (32). Ålgars et al. (33) found that transgender persons try to conceal the characteristics of their assigned sex or demonstrate the characteristics of their required gender identity through changes in dietary behavior. The common gender stereotypes of parents often teach boys to change their feminine characteristics or behaviors, and poor teaching may increase boys’ psychological burden and unhealthy eating behaviors (34). Sociocultural pressure may increase the risk of disordered weight control behaviors (such as unhealthy eating behaviors) for gender-non-conforming girls (35). Adolescents with non-conforming gender expression in school and online environments are more likely to experience peer bullying and peer violence (36, 37) and may have an increased risk for disordered weight control behaviors (38). The greater the social pressure of children who want to be of the opposite gender, the more serious unhealthy eating behaviors may be (11).

In addition, we found that boys who disliked their own gender had more protein eating behaviors. Studies have shown that boys are more likely to have strong muscles, while girls are more likely to have slim bodies (39). Muscle strength is an important determinant of physical performance in children and adolescents (40). Boys who do not like their gender might feel that way because they are not strong enough and thus hope to become strong through higher protein eating behavior (41). Men’s pursuit of muscle is also an internal social and cultural pressure, which suggests that men should have a muscular body (42). However, we found that there were not more protein eating behaviors among boys who wanted to be the opposite gender, and there were no differences in healthy eating behaviors between boys who wanted to be the opposite gender and girls who did not, which shows that boys who want to be the opposite gender may not make themselves stronger, but their goal may be to make themselves more like girls through healthy eating behaviors. Studies have shown that boys who want to be the opposite gender still want to have large hips and breasts despite their healthy weight (43). Homosexual men pay more attention to thin bodies (11). We found that girls who disliked their own gender or who wanted to be the opposite gender had higher protein eating behavior, and there was no difference from boys who liked their gender or did not want to be the opposite gender. Girls who did not like their gender might not be slim enough, but they wanted to make themselves into boys through higher protein eating behaviors, which is in line with the femininity hypothesis (44). In addition, the results of multiple linear regression showed that girls who wanted to be of the opposite gender had fewer unhealthy eating behaviors than girls who did not want to be of the opposite gender, which shows that girls with this desire are more motivated to develop toward the opposite sex through eating behaviors. We found that there was a positive interaction between disliking one’s own gender and wanting to be the opposite gender in boys’ midnight snack eating and in girls’ consumption of carbonated drinks, fish, meat, and shrimp and their protein dietary factors, which indicates that if a child does not like their own gender and has the desire to become the opposite gender at the same time, the above eating behaviors will be more frequent.

This study has several limitations. First, based on a cross-sectional study, we found relationships between negative gender attitudes and eating behaviors; however, whether the correlations are causal remains to be verified by cohort studies. Second, the children and adolescents surveyed were from two primary and secondary schools in China, which has limitations in extending other adolescents. Finally, all the collected data were the participants’ self-reports, and their responses may be affected by factors such as denial, idealization and social expectations.

## Conclusion

The proportion of girls with a negative gender attitude (disliking their own gender or wanting to be the opposite gender) was higher than that of boys. Boys who disliked their own gender or wanted to be the opposite gender may have higher frequencies of unhealthy eating behaviors and lower frequencies of healthy eating behaviors. Girls who disliked their own gender or wanted to be the opposite gender may have higher frequencies of protein eating behaviors. While encouraging children to develop healthy eating behaviors, we should pay attention to children’s positive attitudes toward their own gender cognition.

## Data availability statement

The raw data supporting the conclusions of this article will be made available by the authors, without undue reservation.

## Ethics statement

The studies involving human participants were reviewed and approved by the Medical Research Ethics Committee of Bengbu medical college [(2015) No. 003]. Written informed consent to participate in this study was provided by the participants’ legal guardian/next of kin.

## Author contributions

RC and JC: conceptualization. KL, YW, XP, and JZ: methodology. RC: formal analysis and data curation. LF, RY, and HH: investigation. RC and LF: writing — original draft preparation and writing — review and editing. MH, RY, and HH: visualization. LF: project administration. All authors contributed to the article and approved the submitted version.
